# Validation of automated DTI-ALPS and free water index as potential survival stratification markers in IDH-wildtype glioblastoma

**DOI:** 10.1007/s00234-026-03928-7

**Published:** 2026-02-06

**Authors:** Minchul Kim, Min Seo Choi, Inpyeong Hwang, Chul-Kee Park, Seung Hong Choi, Kyu Sung Choi

**Affiliations:** 1 Department of Radiology, Kangbuk Samsung Hospital, Sungkyunkwan University School of Medicine, Seoul, Korea, Republic of; 2 Department of Radiology, Seoul National University Hospital, Seoul, Korea, Republic of; 3 Department of Neurosurgery, Seoul National University Hospital, Seoul, Korea, Republic of; 4 Department of Radiology, Seoul National University College of Medicine, Seoul, Korea, Republic of

**Keywords:** DTI-ALPS, Neurofluid dynamics, Glioblastoma, Survival, Free water

## Abstract

**Purpose:**

Diffusion MRI–based indirect indices of neurofluid dynamics, such as diffusion tensor imaging along the perivascular space (DTI-ALPS) and extracellular free water (FW), have been reported to have prognostic implications in glioblastoma. However, their clinical utility, replicability, and the relation between the tumor burden remain insufficiently investigated with inconsistent results hindering its integration into routine imaging.

**Methods:**

Two datasets of isocitrate dehydrogenase-wildtype (IDHwt) glioblastoma were retrieved from The Cancer Imaging Archive (UPENN = 200, UCSF = 125). The automated DTI-ALPS (aDTI-ALPS) and the FW index was used as an indirect indicator of glymphatic function. Prognostic significance was assessed using Kaplan–Meier analysis along with log-rank test, and multivariable Cox regression including aDTI-ALPS and clinical variables such as age, and extent of resection. Associations between MRI indices and tumor characteristics were also evaluated.

**Results:**

A higher aDTI-ALPS index was associated with longer survival (*P =* 0.024 and 0.018 for both datasets, respectively; log-rank test), indicating its prognostic significance. Multivariable Cox analysis revealed low aDTI-ALPS as an independent factor for poor prognosis (hazard ratio (HR), 1.352; *P =* 0.050 and hazard ratio, 1.616; *P =* 0.044, respectively). Low FW index was associated with longer survival in UPENN. Additionally, the aDTI-ALPS index showed no correlation with tumor volume or tumor laterality, and the HR was smaller than molecular markers.

**Conclusion:**

Automated DTI-ALPS and the free water index may serve as reproducible prognostic factors in IDH-wildtype glioblastoma, while showing no significant correlation with tumor burden.

**Supplementary Information:**

The online version contains supplementary material available at 10.1007/s00234-026-03928-7.

## Summary section

**Previous literature:**

Neurofluid dynamic, measured by diffusion MRI indices such as DTI-ALPS and extracellular-free water, has been reported to have prognostic implications for glioblastoma. However, their clinical utility, replicability, and the relation between the tumor burden remain insufficiently investigated with inconsistent results.

**Key findings:**

The automated DTI-ALPS index is an independent prognostic marker for IDH-wildtype glioblastoma regardless of acquisition parameters. Additionally, it consistently showed no association with tumor volume or laterality, suggesting that it may reflect underlying pathophysiological mechanisms distinct from direct mechanical compression or mass effect. Meanwhile, the prognostic performance is modest, with less significant effect than MGMT methylation status and extent of resection, indicating questions remain to be addressed to have clinical utility.

## Introduction

Isocitrate dehydrogenase (IDH)-wildtype glioblastoma is the most common primary brain tumor in adults, accounting for approximately 50% of all malignant brain tumors [1]. Around the late 2000s, advanced molecular diagnostics led to reports of IDH mutation in glioblastomas, serving as a defining branch point [1, 2]. It became more evident over the last decade that glioblastomas with IDH mutation are biologically distinct from IDH-wildtype glioblastomas with a longer median overall survival (OS) of approximately 3.6 years [3], and the term “glioblastoma” is no longer applied to CNS World Health Organization grade 4 IDH-mutant astrocytoma [4]. Therefore, exploring an effective prognostic biomarker of IDH-wildtype glioblastoma is crucial.

The so-called glia-lymphatic or glymphatic system has been recently acknowledged as a brain waste clearance system. Recently, several established MRI-based noninvasive methods, including calculation of the fractional volume of free water (FW) in brain parenchyma (i.e., brain interstitial fluid) from a bi-tensor diffusion tensor imaging (DTI) model [5], and the diffusion along perivascular spaces (DTI–ALPS) index [6], were introduced to indirectly assess neurofluid dynamics in vivo using MRI [7–10]. Elevated white matter (WM) FW has been reported in patients with Alzheimer disease, suggesting the stagnation of interstitial fluid drainage throughout the brain [11]. Using the DTI-ALPS index, researchers have significantly advanced our understanding of neurofluid dynamics in brain tumors [7–10, 12, 13]; brain tumor patients consistently exhibit significantly lower DTI-ALPS indices compared to healthy controls, indicating impaired glymphatic function. This is thought to attributed to tumor-induced compression of perivascular spaces and altered fluid dynamics [10]. Follow-up studies focused on brain glioma reported the reduced DTI-ALPS in glioma patients, and the DTI-ALPS index may be used as a potential biomarker for glioma grading, IDH mutation status prediction [9], or even serve as a prognostic factor for high-grade glioma [8].

However, prior studies have reported inconsistent findings regarding the association between the DTI-ALPS index measurement methods and tumor characteristics (Table 1). For instance, while Gao et al. observed a strong negative correlation between the DTI-ALPS index and glioblastoma tumor volume (*r* = − 0.786), Wang et al. found no significant relationship between tumor volume and either ipsilateral DTI-ALPS index in patients with high-grade glioma (Table 1, fifth row) [8, 10]. Similarly, the association between tumor laterality and DTI-ALPS has varied across studies: Zeng et al. reported decreased DTI-ALPS values on the tumor side, Wang et al. observed higher tumor-side values, and Uchida et al. found no laterality-based difference (Table 1, sixth row) [7–10, 12, 13]. These discrepancies may be partially attributable to reliance on varied size and manual region-of-interest (ROI) placement in conventional DTI-ALPS index (Table 1, second row), which introduces operator-dependent variability and reduces measurement reproducibility.

**Table 1:** Diffusion MRI biomarkers of glymphatic dysfunction in subjects with glioblastomas

Tumor type (*n*)	Gao et al.	Wang et al.	Zhu et al.	Zeng et al.	Uchida et al.
	Histopathological GBM (30)	WHO 2021 grade IV glioma (experiment set (UCSF),177; validation set (UPENN),59)	Glioma (total 81; 36 GBM)	Glioma (total 91; 44 IDHwt)	IDH wild-type glioblastoma (UPENN), (277)
DTI-ALPS ROI placement (ROI definition)	Manual (3-mm cubic ROIs)	Manual (circular ROIs)	Manual (Circular ROIs with a 5-mm diameter)	Manual (spherical ROIs 5 mm in diameter)	Manual (spherical ROIs 4 mm in diameter)
B value (seconds/mm^2^)	1000	experiment set,2000; validation set,1000	1250	1000	1000
FW evaluation	-	-	-	-	Cox Hazard ratio of high normal side FW (Multivariate, age, sex, tumor volume): 1.34*
Tumor/edema volume and DTI-ALPS correlation (r)	Tumor: (-0.786*) Edema: (-0.078)	Tumor (-0.16*, normal side) Edema (-0.24*, normal side) Tumor side DTI- ALPS (-)	-	Tumor (− 0.002*, normal side) Edema (− 0.002*, normal side)	-
Tumor laterality and DTI-ALPS	-	Tumor side was significantly larger in two datasets (experimental, 1.34 vs. 1.26*; validation, 1.37 vs. 1.25)	Tumor side was significantly lower (1.41 vs. 1.47*)	Tumor side was significantly lower in all tumor types (1.37 vs. 1.40*)	No significant difference between ipsilateral and contralateral normal appearing white matter
Cox Hazard ratio of DTI-ALPS	-	Low difference of ALPS-index group (Univariate): 1.92*	-	Normal side of ALPS-index (Univariate): 0.095*	Normal side of ALPS-index (Multivariate, age, sex, tumor volume): 0.71*

*DTI-ALPS* diffusion tensor image analysis along perivascular spaces;

^*^indicates statistical significance of *P* < 0.05

-not reported

**Table 2:** Clinical characteristics of the entire study population and high versus low aDTI-ALPS groups

(A) Baseline clinical and imaging characteristics of the UPENN (*n* = 200) and UCSF (*n* = 125) cohorts			
	UPENN (*n* = 200)	UCSF (*n* = 125)	*P*-value
Age (years)	63.38 ± 11.93	60.09 ± 12.49	0.018^*^
Gender (Male: Female)	130:70	71:54	0.14
aDTI-ALPS	1.25 ± 0.19	1.08 ± 0.17	< 0.001^*^
Free water	0.014 ± 0.007 (*n* = 408)	0.024 ± 0.008 (*n* = 328)	< 0.001^*^
EOR (STR: GTR)	77:123	31:94	0.011^*^
(B) Comparison of clinical characteristics between patients with high and low aDTI-ALPS in the UPENN cohort.			
	UPENN		*P*-value
	High aDTI-ALPS (*n* = 92)	Low aDTI-ALPS (*n* = 108)	
Age (years)	61.23 ± 12.49	65.21 ± 11.17	0.018^*^
Gender (Male: Female)	48:44	86:22	< 0.001^*^
aDTI-ALPS	1.42 ± 0.14	1.11 ± 0.11	< 0.001^*^
EOR (STR: GTR)	32:60	45:63	0.319
(C) Comparison of clinical characteristics between patients with high and low aDTI-ALPS in the UCSF cohort			
	UCSF		*P*-value
	High aDTI-ALPS (*n* = 55)	Low aDTI-ALPS (*n* = 70)	
Age (years)	59.00 ± 12.72	60.95 ± 12.34	0.387
Gender (Male: Female)	30:25	41:29	0.652
aDTI-ALPS	1.25 ± 0.11	0.94 ± 0.064	< 0.001^*^
EOR (STR: GTR)	14:41	17:53	0.881

*aDTI-ALPS* automated diffusion tensor image analysis along perivascular spaces; *EOR* extent of surgical resection; *STR* subtotal resection; *GTR* gross total resection. Note that both the UPENN and UCSF datasets are the subsets comprising only IDH-wildtype glioblastomas from the original UPENN-GBM and UCSF-PDGM datasets

^*^P < 0.05 indicates statistical significance

To address this, automated ROI placement (aDTI-ALPS) has been proposed to reduce inter-rater variability and enable consistent analysis, especially in large-scale or multi-institutional studies [14–17]. However, despite growing interest in glymphatic imaging, no studies have utilized aDTI- ALPS focused on IDH-wildtype glioblastoma — a clinically aggressive and molecularly homogeneous group in urgent need of reliable prognostic imaging biomarkers.

Here, using two large publicly available IDH-wildtype glioblastoma datasets, we investigated whether the reproducible glymphatic indices, including aDTI-ALPS and FW index, obtained using an automated pipeline, may serve as surrogate prognostic markers of IDH-wildtype glioblastoma and how the metrics are related to tumor volume compositions and tumor laterality.

## Materials and methods

### Study population

**Fig. 1:**
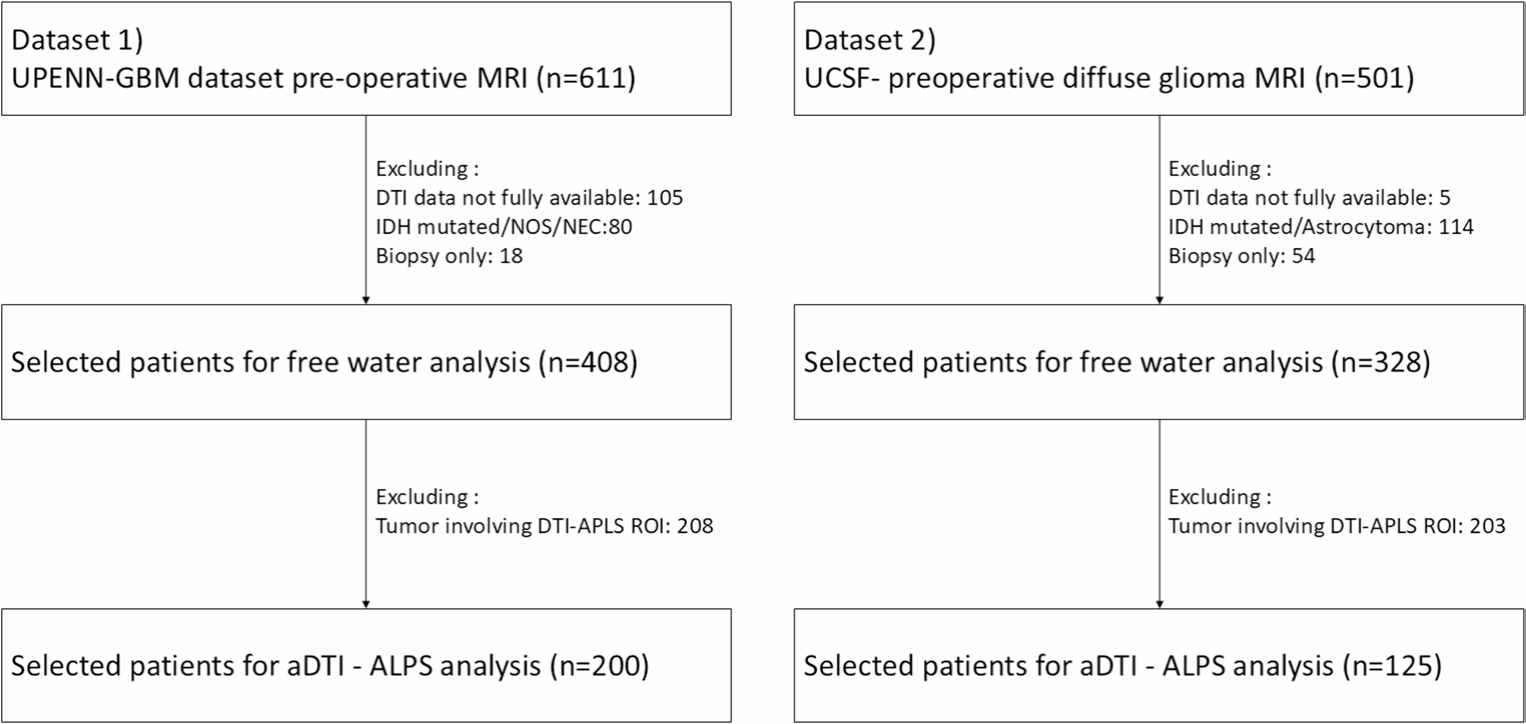
The flow diagram of data acquisition. GBM, Glioblastoma; DTI, diffusion tensor image; IDH, isocitrate dehydrogenase; aDTI-ALPS, automated diffusion tensor image analysis along perivascular spaces

In this retrospective study, we collected the two largest public datasets for IDH-wildtype glioblastoma imaging data from The Cancer Imaging Archive (TCIA) database: the Multi-parametric magnetic resonance imaging scans for de novo glioblastoma (GBM) patients from the University of Pennsylvania Health System cohort (UPENN-GBM [18], *n* = 611) and the University of California San Francisco Preoperative Diffuse Glioma MRI (UCSF-PDGM [19], *n* = 501) were used for replication analysis. Patients included in UPENN-GBM study were treated according to the standard of care, which included maximal safe resection, radiotherapy, and concomitant and adjuvant chemotherapy with Temolozolomide. All patients underwent MRI scanning before the surgery and tumor genetic testing. Among large glioma subjects, we only included grade 4 IDH-wildtype glioblastoma, according to WHO 2021 CNS tumor classification, from the UPENN-GBM and UCSF-PDGM datasets for subsequent analysis (hereafter referred to as UPENN and UCSF, respectively). The exclusion criteria were as follows (Fig. 1): (1) unavailable DTI sequence in Neuroimaging Informatics Technology Initiative (NIfTI) file format ; (2) biopsy analysis only; (3) tumor involving the ROIs in the projection/association fibers for DTI-ALPS analysis [8]. The images were already preprocessed, brain-extracted, normalized, and tumor subregions were segmented using a fully automatic method trained on radiologist-drawn tumor masks [18].

### DTI data acquisition parameters in the datasets

All MR images were acquired using the 3.0 T MR scanners. The DTI data from UPENN dataset was acquired with the following scan parameters: TR = 4200–6300 ms, TE = 83–104 ms, FOV = 128 × 128 mm, slice thickness = 2.5–5.0 mm, orientation = axial, b = 1000 s/mm2, 90 diffusion weighting gradient directions. The DTI data from UCSF dataset was acquired with the following scan parameters: TR = 8400 ms, TE = 73 ms, FOV = 280 × 280 mm, slice thickness = 2.0 mm, orientation = axial, b = 2000 s/mm2, 55 diffusion weighting gradient directions.

### aDTI-ALPS index calculation

**Fig. 2:**
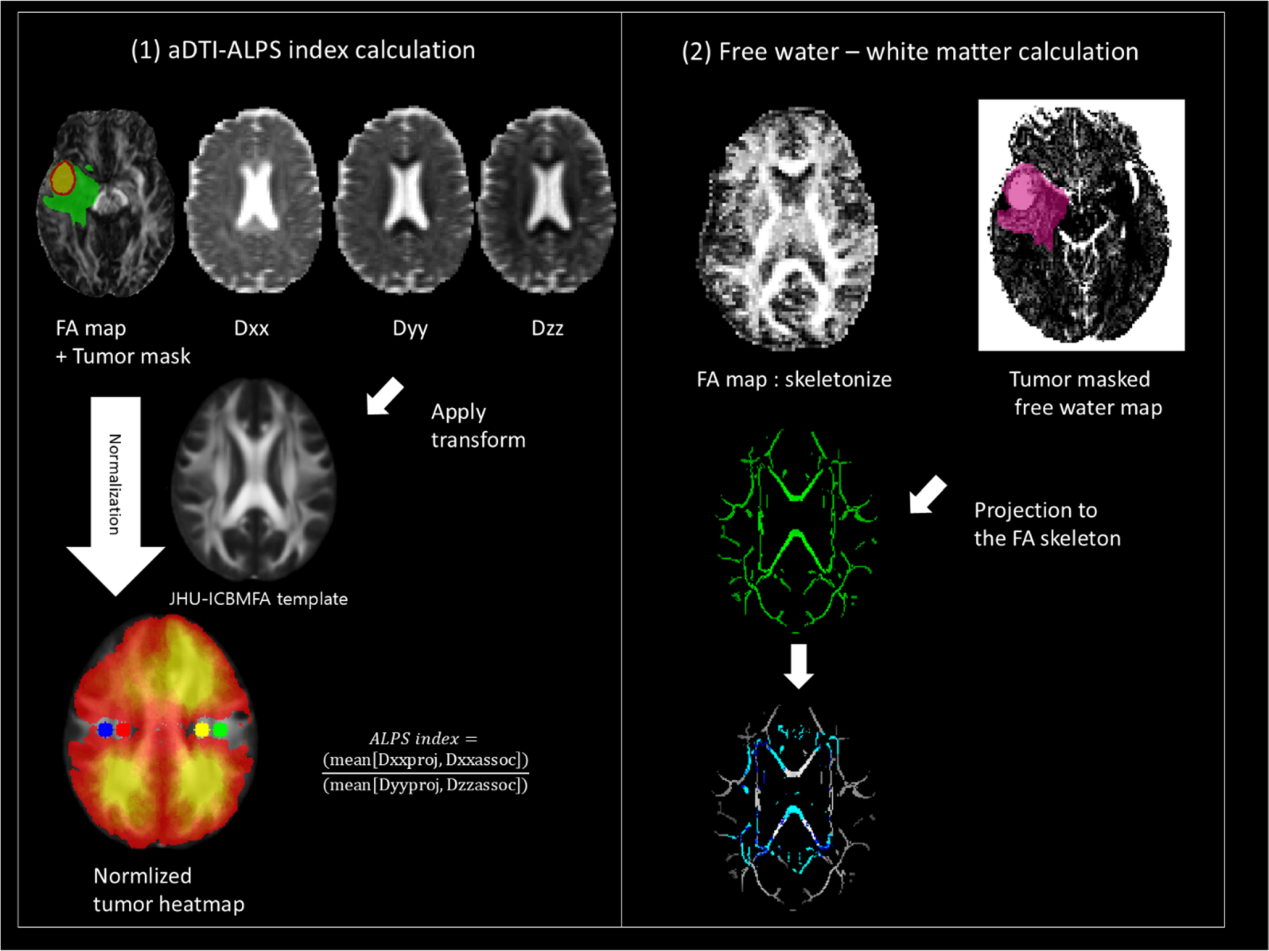
Schematic diagram of the study flow. Diffusion-weighted images were obtained from all study participants and preprocessed. The preprocessed images were then used to calculate the (1) automated diffusion along perivascular spaces (aDTI-ALPS) index and (2) white matter extracellular fractional volume of free water (FW). The aDTI-ALPS index was calculated as follows: The FA map of each subject was co-registered to the JHU-ICBM-FA template, and the transformation matrix was applied to all the diffusivity maps and tumor segmentation maps. The tumor segmentation superimposed on a FA map depicts the sub-region labels: enhancing tumor core (red), necrotic/cystic core (yellow), and peritumoral edematous/infiltrated tissue (green). The pre-defined ROIs for projection (superior corona radiata, blue and green blue) and association fibers (superior longitudinal fasciculus, red and yellow) at the level of lateral ventricle body were used to obtain the diffusivity values of Dxxproj, Dxxassoc, Dyyproj and Dzzassoc (image on the right). If the tumor involved ROIs, then the subject was excluded from the survival analysis (tumor heatmap). The FW was calculated as follows: a FW map was constructed based on a bi-tensor model and the tumor portion was removed; a white matter skeleton was constructed using a tract-based spatial statistics pipeline; and the FW values were normalized to the WM skeleton. The FW values were averaged, using the white matter skeleton as the mask

We used an automated method to calculate the DTI-ALPS index to improve replicability and reliability, which is available at: https://github.com/gbarisano/alps [20]. The DTI images underwent artifact corrections using Marchenko-Pastur Principal Component (MP-PCA) denoising algorithm and Gibbs unringing using MRtrix3 command line “dwisenoise” and “mrdegibbs”, corrections of eddy currents and movements were accomplished with FSL command line “eddy” [21]. After, the fractional anisotropy (FA) map and x-, y- and z-axis diffusivity maps were generated using FSL command line “dtifit”. The FA map of each subject was co-registered to the JHU-ICBMFA template and the transformation matrix was applied to all the diffusivity maps by using FSL command line “flirt”. The projection and association fibers at the level of lateral ventricle body were recognized as the superior corona radiata (SCR) and the superior longitudinal fasciculus (SLF) based on the JHU-ICBM-DTI-81-white matter Labeled Atlas and the ROIs were automatically defined as spheres with 2.5 mm radius in the areas of bilateral projection fibers (superior corona radiata, Fig. 2-(1), blue and green ROIs) and association fibers (superior longitudinal fasciculus, Fig. 2-(1), red and yellow ROIs) which applied on all subjects’ diffusivity maps. The diffusivity values of Dxx, Dyy and Dzz of bilateral SLF and SCR were automatically outputted for the DTI-ALPS index calculation [20]. If the tumor involved the ROIs in the projection/association fibers, the subject was excluded from subsequent analysis (Fig. 2-(1), tumor heatmap). The DTI-ALPS index is defined by the average of bilateral DTI-ALPS indexes (mean DTI-ALPS index), which is the ratio of the mean of x-axis diffusivity in the area of projection fibers (Dxxproj) and x-axis diffusivity in the area of association fibers (Dxxassoc) to the mean of the y-axis diffusivity in the area of projection fibers (Dyyproj) and z-axis diffusivity in the area of association fibers (Dzzassoc) as follows [6]:

$$\:\mathrm{D}\mathrm{T}\mathrm{I}-\mathrm{A}\mathrm{L}\mathrm{P}\mathrm{S}\:\mathrm{i}\mathrm{n}\mathrm{d}\mathrm{e}\mathrm{x}=\:\frac{\mathrm{m}\mathrm{e}\mathrm{a}\mathrm{n}\left(\mathrm{D}xxproj,\:\mathrm{D}xxassoc\right)}{\mathrm{m}\mathrm{e}\mathrm{a}\mathrm{n}(\mathrm{D}yyproj,\:\mathrm{D}zzassoc)}$$

### Free water calculation

FW-corrected DTI maps were calculated using an in-house MATLAB script provided by the authors of Bergamino et al. The FW maps were computed by fitting the following 2-compartment model at each voxel [22–26].

$$\:{\mathrm{A}}_{\mathrm{g}}\left(\mathrm{D},\mathrm{f}\right)=\mathrm{f}\cdot\:\:\left(\mathrm{e}\mathrm{x}\mathrm{p}\left[-\mathrm{b}{\mathrm{g}}^{\mathrm{T}}\mathrm{D}\mathrm{g}\:\right]\right)+\left(1-f\right)\cdot\:exp\left[-b{D}_{water}\right]$$

The FW map represents the relative fraction of FW in each voxel, ranging from 0 to 1. To calculate the FW in white matter, we adopted a procedure from tract-based spatial statistics [27]. The FA data from all subjects were aligned to the 1 × 1 × 1 mm³ FMRIB58_FA standard space atlas via nonlinear registration. Subsequently, a mean FA image was generated from the aligned FA images and thinned to form a skeletonized mean FA image representing the central axes of all tracts common to all subjects. The mean FA skeleton was thresholded to FA ≥ 0.2, which is the default value, and each subject’s aligned FA data were projected onto the skeleton. Finally, we acquired the mean of the FW index of each participant’s white matter skeleton using the ‘tbss_non_FA’ and ‘fslmeants’ commands, while the tumor segmentation was used as an exclusion mask [25, 28] (Fig. 2-(2)).

### Statistical analysis

Statistical analyses were performed in JASP [29]. Demographic data of the two groups were compared using either Fisher’s exact test or the Student’s t-test. The primary objective of this study was to determine whether the MRI-based indirect neurofluid dynamic measures could be a marker for survival in IDH-wildtype glioblastoma. Accordingly, the OS was analyzed by the Kaplan-Meier (KM) method and compared by using the Peto and Peto variation of the log-rank test statistic [30]. To account for potential confounding by EOR, we repeated Kaplan–Meier analyses within GTR and STR subgroups. A multivariable Cox proportional hazards regression was performed using variables of age, extent of resection (EOR), and DTI-ALPS to investigate the independent prognostic factors of IDH-wildtype glioblastoma. Regarding the extent of resection (EOR), only patients who underwent either gross total resection (GTR) or subtotal resection (STR) were included. The hazard ratio (HR) with 95% confidence interval (CI) was calculated. The levels of DTI-ALPS or FW index FW index were determined by the mean values, since previous studies tend to used the mean value as the representative value when reporting DTI-ALPS index [31]. The prognostic performance of the survival model was evaluated via the Harrell C index, calculated using the ‘survival’ R package [32]. C-index values of less than 0.6, 0.6–0.7, and greater than 0.7 for the prognostic models were considered poor, moderate, and good, respectively [33]. The prognostic accuracy of the aDTI-ALPS index and FW index was assessed using the data from the UCSF dataset for external validation. As our main prespecified analysis focused on overall survival in the UPENN cohort with aDTI-ALPS as the primary biomarker, and given the exploratory nature of the additional analyses, *P* < 0.05 was considered statistically significant, in line with prior glioblastoma studies using DTI-ALPS [9, 13]. Finally, we sought to investigate the relation between the tumor and the aDTI-ALPS index in both datasets. The correlation analysis was performed between the aDTI-ALPS index and tumor-related volumes (i.e. peritumoral edema volume, tumor volume, and tumor + edema volume), and the aDTI-ALPS index of the tumor side versus the normal side was compared using a paired *t*-test.

We conducted two sensitivity analyses; (1) we calculated the aDTI-ALPS index using ROIs defined as spheres with a 5 mm radius and calculated the correlation between the original aDTI-ALPS, as well as survival analysis, (2) we applied ComBat harmonization (https://github.com/Jfortin1/ComBatHarmonization) to aDTI-ALPS and FW values across sites and repeated the survival analyses using the harmonized metrics (Figure S1) [34], (3) we selected those subjects whose O6-methylguanine-DNA methyltransferase status (MGMT), used as a prognostic/predictive marker in patients with GBM [35], available and included in the Cox multivariate regression model to compare the HR against the diffusion MRI biomarkers of neurofluid dynamics.

## Results

### Patient characteristics

A total of 325 patients with IDH-wildtype glioblastoma were included in the final analysis, comprising 200 patients from the UPENN cohort and 125 from the UCSF cohort. The mean age was slightly higher in the UPENN cohort compared to the UCSF cohort (63.4 ± 11.9 vs. 60.1 ± 12.5 years, *P* = 0.018), with no significant sex difference between cohorts (*P* = 0.14) (Table 2A). The mean aDTI-ALPS index was significantly higher in the UPENN cohort than in the UCSF cohort (1.25 ± 0.19 vs. 1.08 ± 0.17, *P* < 0.001). Similarly, the free water index was significantly lower in the UPENN cohort (0.014 ± 0.007 vs. 0.024 ± 0.008, *P* < 0.001). Regarding the extent of surgical resection, a higher proportion of patients in the UPENN cohort underwent subtotal resection compared to the UCSF cohort (38.5% vs. 24.8%, *P* = 0.011). The tumor distributions of each dataset are displayed in Figure S2, showing mild right dominance in UPENN, while mild left dominant distribution in UCSF dataset. We performed a visual quality assurance (QA) assessment of normalization in randomly selected cases from both the UPENN and UCSF cohorts. In all reviewed subjects, the transformed enhancing tumor, necrotic core, and peritumoral edema masks followed the expected anatomy. Representative examples from cases with extensive edema are shown in Figure S3.

### Multivariable survival analysis: DTI-ALPS index

**Fig. 3:**
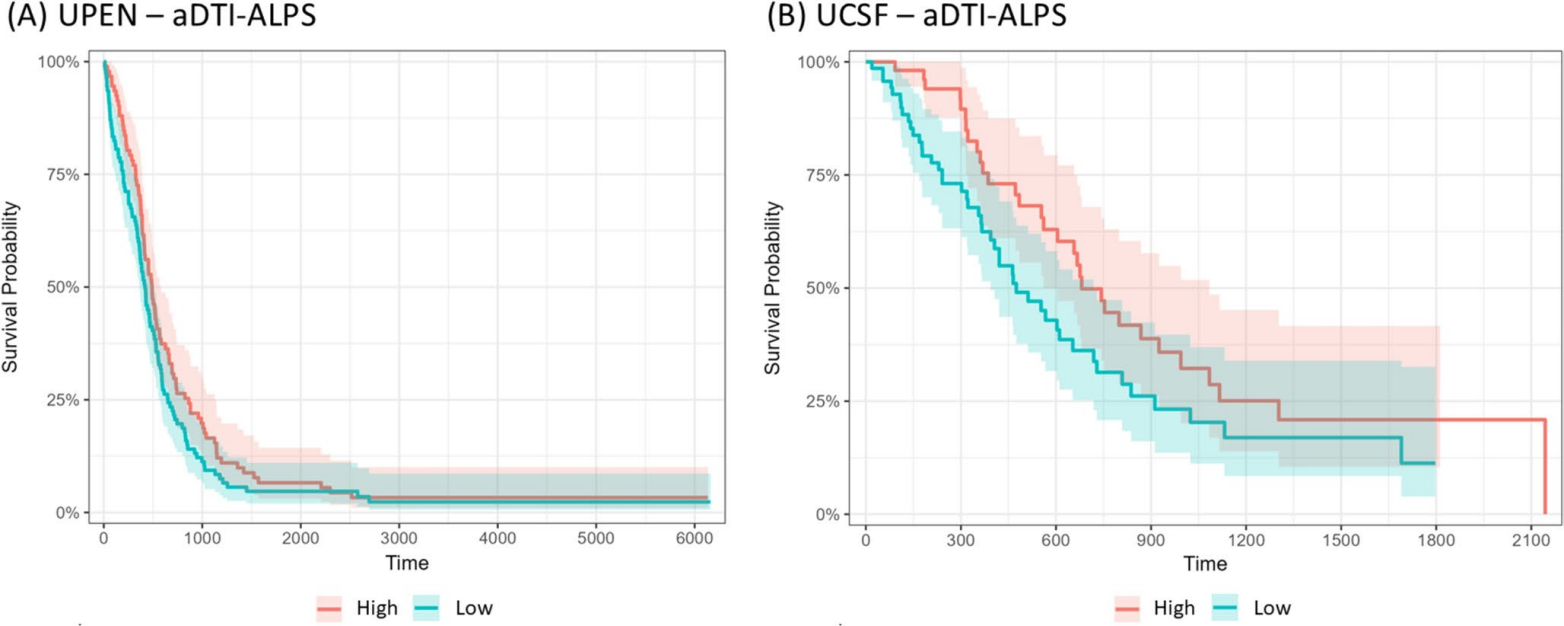

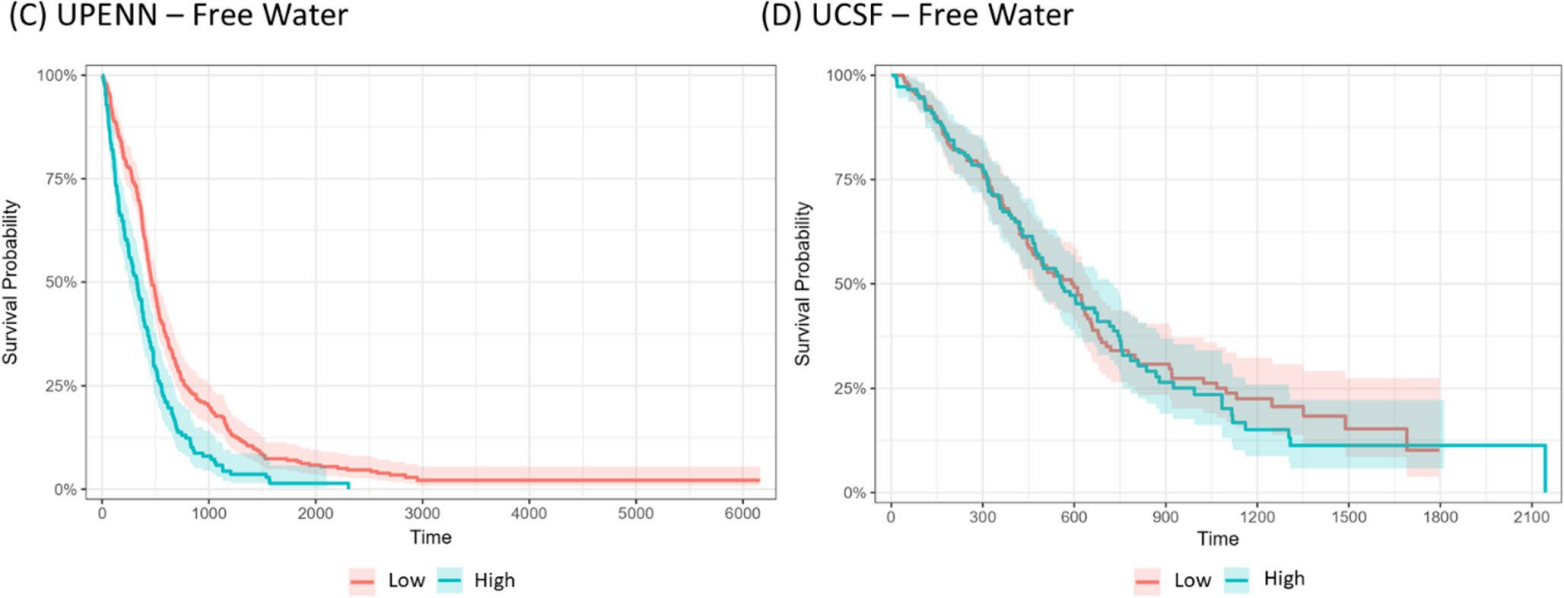
Prognostic significance of the glymphatic indices in the UPENN and UCSF datasets. Panels **A** and **B** show Kaplan–Meier survival curves based on the levels of the DTI-ALPS index in UPENN and UCSF datasets, respectively. Patients with a high aDTI-ALPS index showed a better prognosis in both datasets (*P =* 0.024 and *P =* 0.018, respectively). Panels **C** and **D** show Kaplan–Meier survival curves based on the levels of white matter free water in the UPENN and UCSF datasets, respectively. While patients with low free water index showed better prognosis in the UPENN dataset, no significant difference in survival between the two levels of free water was detected in the UCSF dataset

In the UPENN dataset, the low aDTI-ALPS group had a median OS of 389 days (95% CI 343–509), which was significantly shorter than the 489 days (95% CI 420–553) of the high aDTI-ALPS group (*P =* 0.024; Fig. 3A). Kaplan–Meier analyses stratified by EOR were discordant between cohorts: in UPENN, the aDTI-ALPS group difference was significant in the STR subgroup (*n* = 77, *P* = 0.004) but not in the GTR subgroup (*n* = 123, *P* = 0.818), whereas in UCSF the opposite pattern was observed, with significance in the GTR subgroup (*n* = 94, *P* = 0.030) but not in STR (*n* = 31, *P* = 0.252). Survival analyses using ComBat-harmonized aDTI-ALPS and FW values were consistent with the main analyses. In the UPENN cohort, the low harmonized aDTI-ALPS group had a median OS of 419 days (95% CI, 363–516), which was marginally shorter than the 489 days (95% CI, 412–565) in the high aDTI-ALPS group (*P* = 0.086). In the UCSF cohort, the low harmonized aDTI-ALPS group had a median OS of 475 days (95% CI, 394–719) versus 681 days (95% CI, 561–1084) in the high aDTI-ALPS group, showing a similar trend (*P* = 0.018).

In the multivariable Cox survival analysis, considering age, subtotal resection, and low aDTI-ALPS showed a significantly increased survival risk of low aDTI-ALPS (Table 3, HR 1.352, *P =* 0.050). Age was also a significant factor for OS (Table 3, HR 1.015, *P =* 0.030). The extent of resection had a marginal p-value of 0.069. In the replication cohort of UCSF dataset, the low aDTI-ALPS group repeated had a OS of 475 days (95% CI 394 − 719), which was significantly shorter than the 681 days (95% CI 561–1084) of the high aDTI-ALPS group (*P =* 0.018; Fig. 3B). In the multivariable Cox survival analysis considering age, subtotal resection, and low aDTI-ALPS showed a significant increased survival risk of low aDTI-ALPS (Table 3, HR 1.616, *P =* 0.044). The age and extent of resection were also significant factors for OS (Table 3, HR 1.022, *P =* 0.036 and HR 1.717, *P =* 0.040, respectively). The C-index for the multivariable model was 0.605 (95% CI: 0.558, 0.652) in the UPENN dataset, and 0.636 (95%CI: 0.562, 0.711) in the UCSF dataset, indicating moderate performance (Table 3). The sensitivity analysis using ROIs defined as spheres with a 5 mm radius showed very high correlation with the original one (*r* = 0.983 for both datasets, *P* < 0.001, Figure S4 upper panels), as well as a significant predictor in the survival analysis (UPENN, *P* = 0.033; UCSF, *P* = 0.018, Figure S4 lower panels).

**Table 3:** Multivariable Cox survival analyses for glymphatic index

Datasets	aDTI-ALPS index				FW index			
	UPENN		UCSF		UPENN		UCSF	
Variables	HR	P value	HR	P value	HR	P value	HR	P value
Age	1.015 (1.001, 1.028)	0.030^*^	1.022 (1.001, 1.043)	0.036^*^	1.016 (1.006, 1.026)	0.001^*^	1.035 (1.021, 1.050)	< 0.001^*^
Extent of resection	0.750 (0.550, 1.023)	0.069	0.582(0.347, 0.975)	0.040^*^	0.676 (0.550, 0.832)	< 0.001^*^	0.533 (0.396, 0.717)	< 0.001^*^
Glymphatic Index	1.352 (1.000, 1.834)	0.050	1.616 (1.012, 2.580)	0.044^*^	1.510 (1.210, 1.884)	< 0.001^*^	0.960 (0.723, 1.274)	0.776
C-index	0.605 (0.558, 0.652)	< 0.001^*^	0.636 (0.562, 0.711)	< 0.001^*^	0.632 (0.600, 0.665)	< 0.001^*^	0.639 (0.593, 0.685)	< 0.001^*^

The numbers in the parentheses represent the 95% confidence interval. The HR for pathological categorial variables are calculated relative to a reference category. * *P* < 0.05 indicates statistical significance. The glymphatic indices refer to the aDTI-ALPS and FW indices

*HR* hazard ratio; *aDTI-ALPS* automated diffusion tensor imaging along the perivascular space; *FW*, fractional volume of extracellular-free water

However, in the subset with available MGMT promoter methylation status (UPENN, *n* = 110; deaths = 107; UCSF, *n* = 122; deaths = 75), aDTI-ALPS was not a significant predictor of overall survival (UPENN, HR 0.987, *P* = 0.954; UCSF, HR 0.907, *P* = 0.512), whereas MGMT methylation remained strongly associated with better survival (UPENN, HR 0.156, *P* < 0.001; UCSF, HR 0.748, *P* = 0.069). The C-index for the multivariable model was 0.666 (95% CI, 0.602–0.729) in the UPENN dataset and 0.636 (95% CI, 0.560–0.712) in the UCSF dataset. When we included an interaction term (MGMT × aDTI-ALPS), the C-index increased only minimally and the interaction term was not significant (C-index 0.676 [95% CI, 0.613–0.740] in UPENN and 0.643 [95% CI, 0.567–0.718] in UCSF).

### Multivariable survival analysis: free water index

The high FW index group had a median OS of 326 days (95% CI 248–389), which was significantly shorter than the 466 days (95% CI 428–528) of the low FW index group in UPENN dataset (*p* < 0.001; Fig. 3C). Kaplan–Meier analyses stratified by EOR repeatedly showed that FW was significantly associated with overall survival in both UPENN subgroups (GTR: *n* = 246, *P* < 0.001; STR: *n* = 162, *P* < 0.001), whereas no association was observed in UCSF in either subgroup (GTR: *n* = 215, *P* = 0.934; STR: *n* = 113, *P* = 0.651). Survival analyses using ComBat-harmonized FW values were also consistent with the main analyses: lower FW remained significantly associated with longer overall survival in the UPENN cohort (*P* < 0.001), but not in the UCSF cohort (*P* = 0.699).

In the multivariate Cox survival analysis considering age, subtotal resection, and high FW index showed significant increased survival risk of high FW index (Table 3, HR 1.510, *P* < 0.001). The age and extent of resection were also significant factors for OS (Table 3, HR 1.016, *P* < 0.001 and HR 1.717, *P* < 0.001, respectively). In the replication cohort of UCSF dataset, high FW index group high FW index group had a median OS of 560 days (95% CI 472 − 729), which was not significantly shorter than the 599 days (95% CI 465–657) of the low FW index group (*P* = 0.809; Fig. 3D). In the multivariate Cox survival analysis considering age and subtotal resection, high FW index did not showed significant increased survival risk of high FW index (HR 0.960, *p* = 0.776), while the age and subtotal resection were significant factors for OS (HR 1.035, *p* < 0.001 and HR 1.877, *P* < 0.001, respectively) (Table 3).

However, in the subset with available MGMT promoter methylation status for the FW analysis (UPENN, *n* = 225; deaths = 219; UCSF, *n* = 311; deaths = 188), a higher FW index was not a significant predictor of overall survival (UPENN, HR 1.600, *P* = 0.954; UCSF, HR 1.423, *P* = 0.497), whereas MGMT methylation remained associated with improved survival (UPENN, HR 0.474, *P* = 0.002; UCSF, HR 0.819, *P* = 0.333). The C-index for the multivariable model was 0.670 (95% CI, 0.626–0.714) in UPENN and 0.638 (95% CI, 0.591–0.686) in UCSF. Including an interaction term (MGMT × FW) led to only a minimal change in C-index, and the interaction term was not significant (C-index 0.671 [95% CI, 0.626–0.715] in UPENN and 0.638 [95% CI, 0.591–0.686] in UCSF).

### Association between the tumor, the aDTI-ALPS index, and free water

**Fig. 4:**
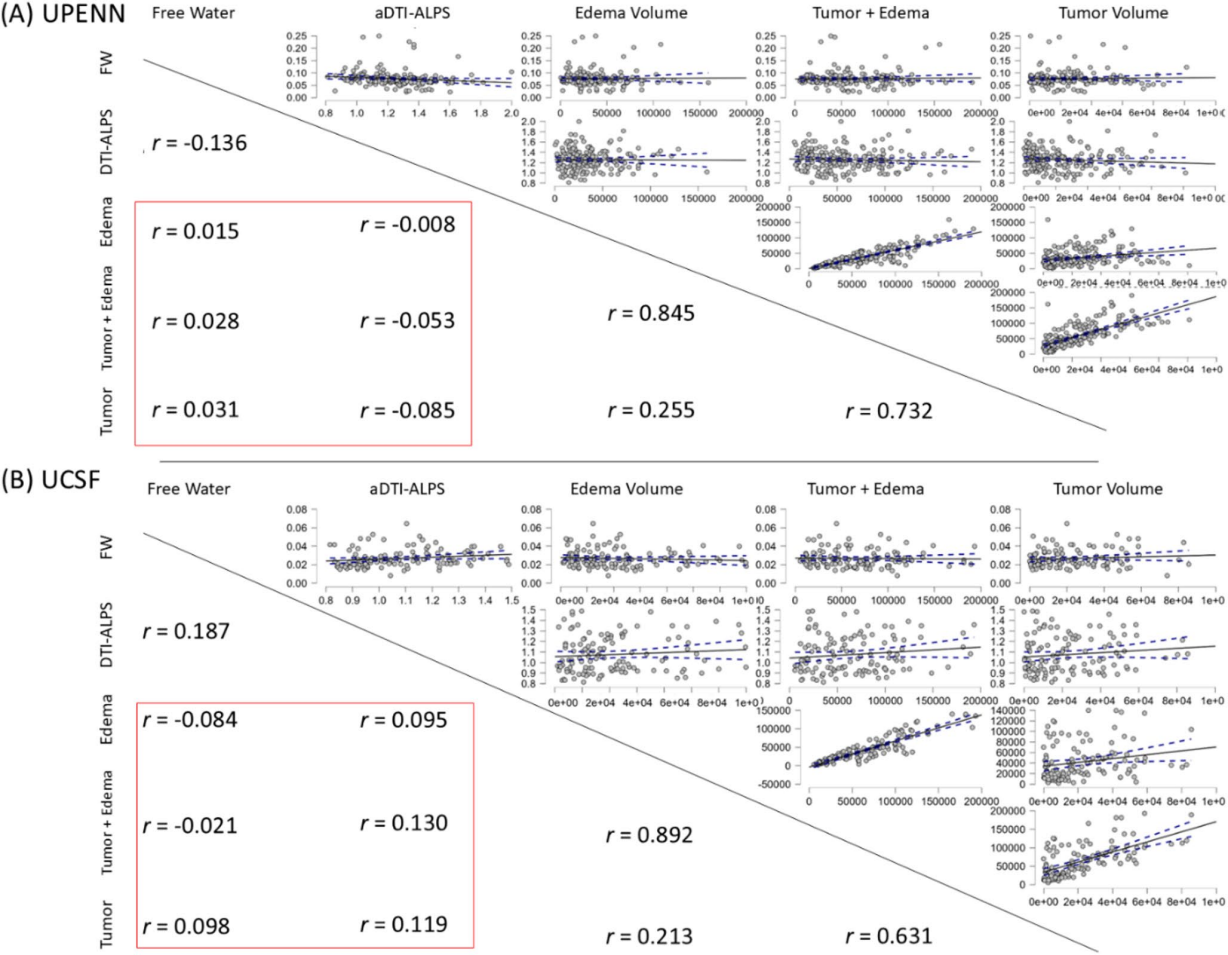
Correlation plots of the glymphatic indices and tumor-related volumes. The lower triangle of Panel **A** and **B** displays the Pearson correlation coefficients between variables, revealing the strength and direction of their correlation. The upper triangle features a scatter plot with fitting lines and their confidence intervals. Notably, there is no significant relationship between the brain tumor volumes and the glymphatic indices including free water and aDTI-ALPS index, for both datasets

Correlation analysis showed no significant association between the aDTI-ALPS index nor FW index and tumor-related volumetric measures, including tumor volume, peritumoral edema volume, and the combined tumor + edema volume in either dataset (Fig. 4, red rectangle). In the UPENN cohort, the Pearson’s correlation coefficients (r) between the aDTI-ALPS index and tumor volume, edema volume, and tumor + edema volume were − 0.085, − 0.008, and − 0.053, respectively (Fig. 4A). Similarly, in the UCSF cohort, the corresponding r-values were 0.119, 0.095, and 0.130, respectively (Fig. 4B). All r-values between the aDTI-ALPS index, FW index and tumor and/or peritumoral edema volume were < 0.15, indicating a small effect size.

**Fig. 5:**
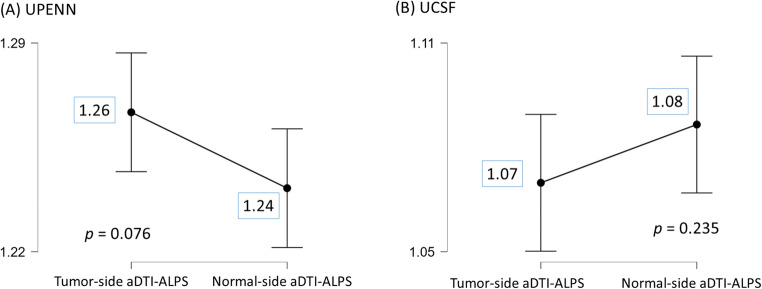
Comparison of glymphatic index based on tumor laterality. Panels **A** and **B** show the aDTI-ALPS-index comparison along the tumor-side and normal-side hemispheres of isocitrate dehydrogenase-wildtype glioblastoma, revealing an opposite result between the UPENN and UCSF datasets

With respect to tumor laterality, the mean aDTI-ALPS index in the hemisphere ipsilateral to the tumor was slightly higher than that of the contralateral side in the UPENN dataset (1.26 vs. 1.24, *P* = 0.076, Fig. 5A). In contrast, the UCSF dataset showed a trend in the opposite direction, with lower tumor-side aDTI-ALPS values compared to the contralateral side (1.07 vs. 1.08, *P* = 0.235, Fig. 5B). However, these differences were not statistically significant in either dataset.

## Discussion

In this study, we aimed to better understand the role of MRI based neurofluid dynamic measures in the prognosis of IDH-wildtype glioblastoma. Using the robustly measured aDTI-ALPS index (Figure S2), we discovered that the aDTI-ALPS index could be useful for risk stratification in IDH-wildtype glioblastoma survival (*P =* 0.024 and 0.018 for UPENN and UCSF datasets; log-rank test, Fig. 3A), which is in line with previous reports utilizing manual ROI drawing [7, 8, 12]. The aDTI-ALPS index was an independent predictor in multivariable Cox regression analysis (HR, 1.352, *P =* 0.050 and HR, 1.616, *P =* 0.044, respectively, Table 3), thereby providing an additional surrogate marker for progression in IDH-wildtype glioblastoma. To our knowledge, our study is the first to demonstrate the prognostic relevance of aDTI-ALPS specifically in IDH-wildtype glioblastoma, using two large publicly available datasets. Specifically, studies by Zeng et al. and Wang et al. investigated the prognostic implications of DTI-ALPS in a mixture of IDH-mutant and wildtype gliomas, while Uchida et al. used manually drawn DTI- ALPS within UPENN dataset [12].

To reduce variability and enhance the utility of DTI-ALPS as a biomarker [36], our study used the automated ALPS ROI approach is less operator-dependent and more scalable than manual delineation, but we acknowledge that manual ROIs can remain the reference for fine-grained anatomical accuracy in individual cases [15]. By employing automated ROI placement and excluding tumor-infiltrated or edematous regions (Fig. 2 and S2, tumor heatmap), we minimized measurement bias according to ROI size (*r* = 0.983 for both datasets, *P* < 0.001, Figure S4 upper panels) [15, 16]. Furthermore, we used the mean of the left and right DTI-ALPS index to reduce noise from any confounders that may affect hemispheric differences of the index. Using the mean DTI-ALPS value computed by the automated pipeline would enhance the efficiency and stability of DTI-ALPS index calculation and the applicable to a large sample, thus enhancing the potential to use as a surrogate survival marker.

Regarding underlying mechanisms of glymphatic alteration in gliomas, two major hypotheses have been proposed to explain glymphatic dysfunction in glioma: first, the glymphatic drainage may be altered due to physical compression by the tumor mass effect [10, 37]. The other hypothesis explaining glymphatic alteration in brain tumors suggests the disruption of the blood-brain barrier and altered interstitial fluid dynamics, which results from the brain white matter infiltration of tumor and inflammatory cells [8–10]. However, previous studies report conflicting results when correlating DTI-ALPS with tumor characteristics (Table 1, fifth row). Such variability may arise from biological heterogeneity or from known interhemispheric confounders such as handedness, language lateralization, and prior COVID-19 infection [38–41], all of which have been reported to influence DTI-ALPS values.

When we correlated the aDTI- ALPS index with tumor burden or tumor laterality to delineate the direct pathophysiological relationship, we did not find a consistent, nor significant relationship (Figs. 4 and 5). In our analysis, laterality results were also inconsistent: the UPENN dataset showed higher DTI-ALPS index values on the tumor side, consistent with Wang et al. [8], while the UCSF dataset showed lower tumor-side values, in line with Zeng et al. (Fig. 5) [7]. These findings suggest that DTI-ALPS asymmetry alone may not reliably reflect tumor-related effects without controlling for systemic and hemispheric factors.

Lastly, we describe some obstacles and limitations to overcome to embrace the aDTI-ALPS or FW as a surroagate marker of survival in IDH-wildtype glioblastoma. First, the prognostic performance of aDTI-ALPS or FW is modest (C-index range in 0.60–0.64) and HR was insignificant when MGMT methylation status is incorporated. This questions the added value or clinical relevance of aDTI-ALPS as a surrogate survival marker. Second, no standardized reference value exists for the DTI-ALPS index. Although the metric is reproducible, its absolute range can vary by MRI protocol, hemisphere, and adjustment methods (Table 2A, third row). DTI-ALPS can even vary within a person when acquired with different b-value with higher b-value tend to have low ALPS value [42]. In our study, for instance, mean aDTI-ALPS values differed significantly between the two datasets, emphasizing the need for standard acquisition protocols [36]. Third, excluding tumors involving the ALPS ROIs may bias the cohort toward smaller tumors and those located in the parietal lobe. To assess potential selection bias from excluding cases with tumor or edema involvement of the ALPS ROIs, we compared patients included in the ALPS analysis with those excluded in both cohorts. In UPENN, excluded patients (*n* = 208) had larger tumor volumes (median 99,889 vs. 50,942 mm³, *P* < 0.001) and edema volumes (median 64,737 vs. 28,808 mm³, *P* < 0.001) than included patients (*n* = 200), whereas age at scan (median 62.7 vs. 64.5 years, *P* = 0.839), overall survival (median 400 vs. 437 days, *P* = 0.172), and extent of resection (GTR 59.1% vs. 61.5%; χ²(1) = 0.15, *P* = 0.699) were similar. In UCSF, excluded patients (*n* = 203) likewise had larger tumor volumes (median 28,526.0 vs. 16,204.0 mm³, *P* < 0.001) and edema volumes (median 66,426.0 vs. 33,031.0 mm³, *P* < 0.001), and were more likely to undergo subtotal resection (STR 40.4% vs. 24.8%; χ²(1) = 7.65, *P* = 0.006), while age (median 63.0 vs. 61.0 years, *P* = 0.106) and overall survival (median 389.0 vs. 406.0 days, *P* = 0.244) did not differ significantly. Together, these analyses indicate that the ALPS analytic cohorts are tend to have smaller tumors and less extensive edema, although survival distributions remain comparable between included and excluded patients. Fourth, while the FW index may offer a more direct estimation of extracellular fluid, its prognostic performance was not consistently replicated, possibly due to technical differences in b-value schemes between datasets. In particular, the UCSF dataset employed a high b-value of 2000 s/mm². As high (> 2000 s/mm^2^) b-values risks non-Gaussian diffusion effects and the tensor model does not account for the non-Gaussian part of the diffusion decay, the analysis has to be performed with more sophisticated models that account for non-Gaussianity [26, 43, 44]. As such, interpretation of FW-related findings in this cohort should be made with caution, as the underlying diffusion acquisition parameters may not have been optimal for free water quantification.

## Conclusions

In summary, we validated that MRI-based automated and reproducible indices of neurofluid dynamics have the potential to serve as additional markers for survival in IDH-wildtype glioblastoma. Our findings also suggest that baseline measurements, rather than tumor-induced alteration, may influence overall survival.

## Supplementary Information

Below is the link to the electronic supplementary material.

Supplementary Material 1

## Data Availability

The datasets analyzed in this study are publicly available in The Cancer Imaging Archive ( [https://www.cancerimagingarchive.net/](https:/www.cancerimagingarchive.net) ).
