# Supplementary material for: Validation of automated DTI-ALPS and free water index as potential survival stratification markers in IDH-wildtype glioblastoma
Source: Neuroradiology. 2026 Feb 6;68(7):1871–83. doi: 10.1007/s00234-026-03928-7 (PMC13407576; doi:10.1007/s00234-026-03928-7)

Supplementary Material

Figure S1. Distributions of aDTI-ALPS (left) and free-water (right) indices before and after ComBat harmonization. Histograms show the original site-specific values for the UPENN (brown) and UCSF (yellow) cohorts and the ComBat-harmonized values pooled across sites (blue). ComBat harmonization reduces inter-site differences in the distributions of both aDTI-ALPS and free water, resulting in a single, more comparable distribution for subsequent analyses.


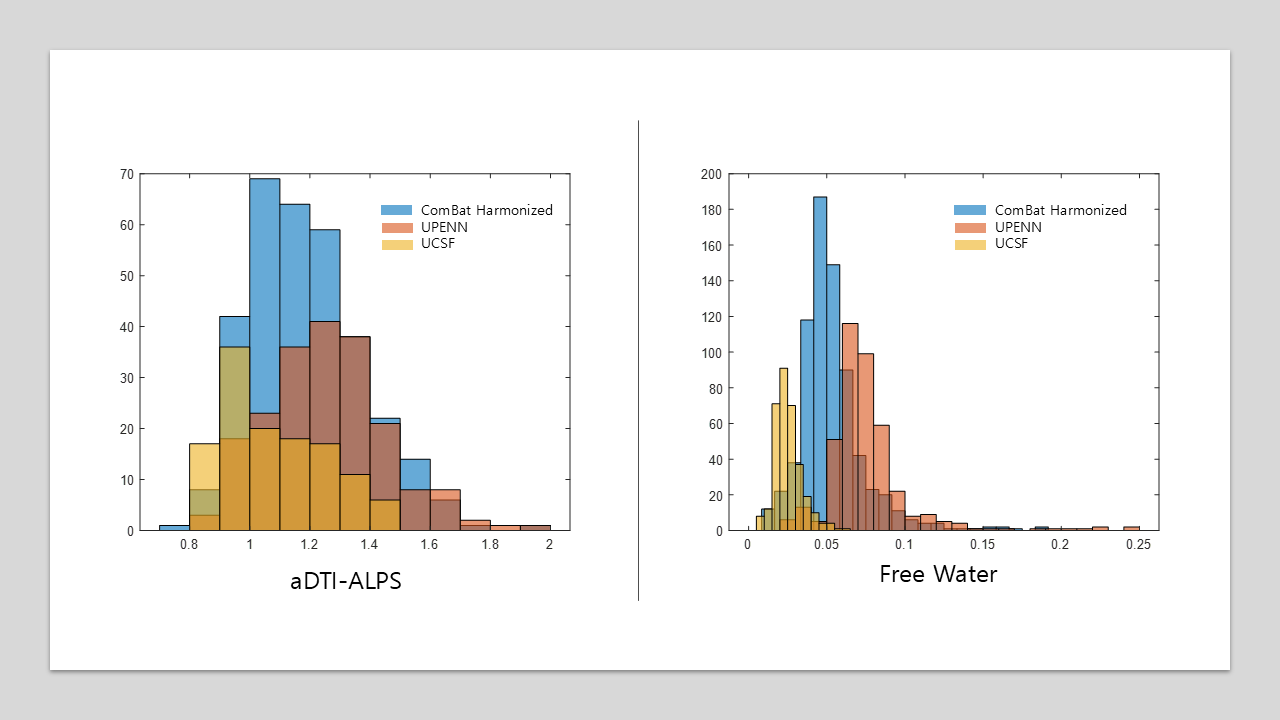


Figure S2. Tumor heatmap showing the major distribution of (A) UPENN and (B) UCSF cohorts.


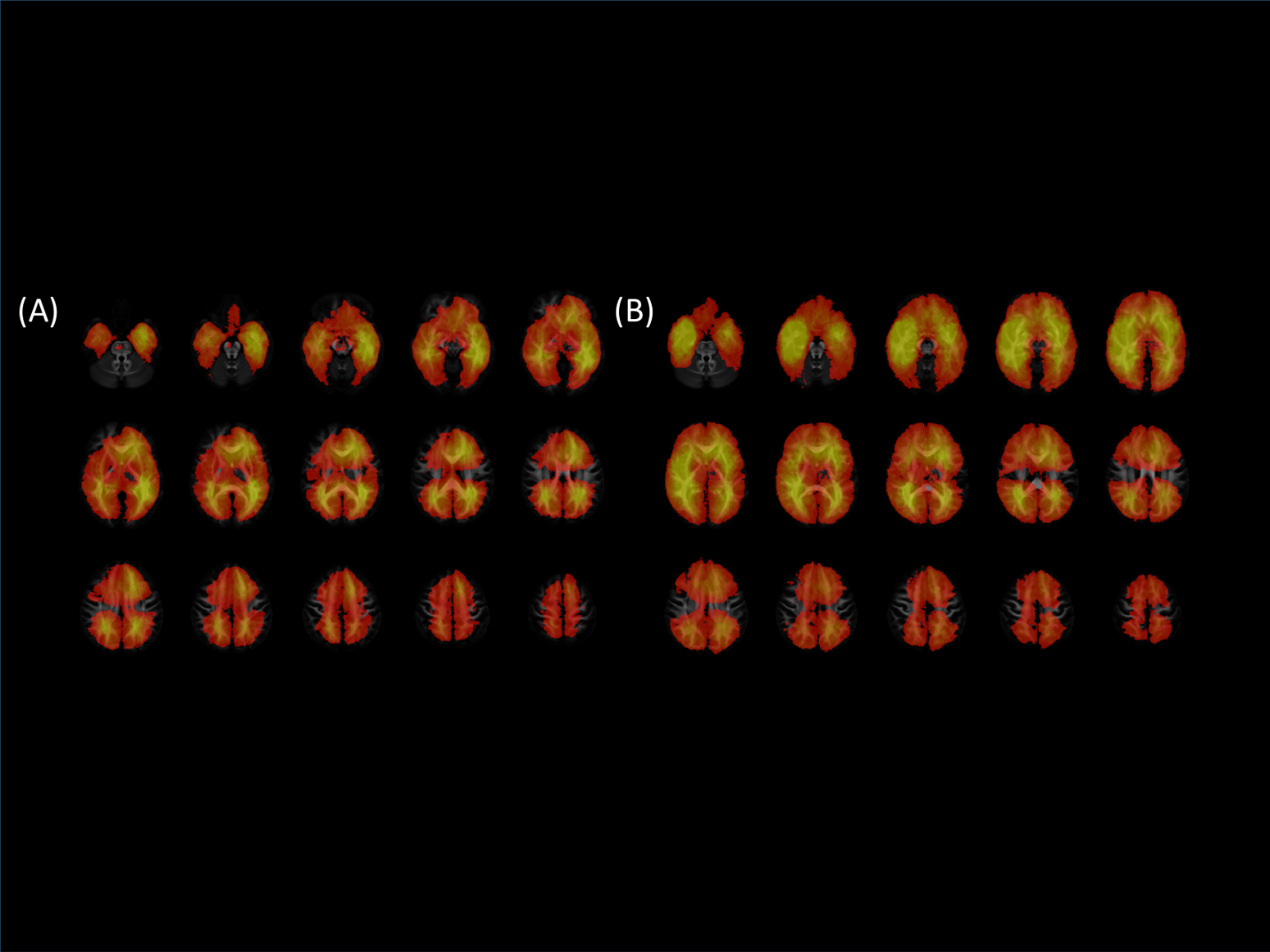


Figure S3. Normalization to MNI space in two representative IDH-wildtype glioblastoma cases with extensive edema from the UPENN (upper case) and UCSF (lower case) cohorts. For each case, the top row shows structural images in the original subject space and the bottom row shows the same images after normalization to MNI space using antsRegistrationSyN. In all images, yellow denotes the enhancing tumor, red the necrotic core, and orange peritumoral edema. In the UPENN case, the blue arrow in the sagittal view highlights edema extending along the corpus callosum, the white arrow in the coronal view marks an enhancing tumor in the superior frontal gyrus, and the green arrow in the axial view indicates edema involving the caudate nucleus and anterior limb of the internal capsule. In the UCSF case, the blue arrow highlights subependymal edema surrounding the occipital horn of the lateral ventricle, the white arrow marks the temporal lobe located tumor mass, and the green arrow indicates edema extending medially adjacent to the thalamus. In both cases, these structures are well preserved in location and shape after transformation, demonstrating acceptable registration quality.


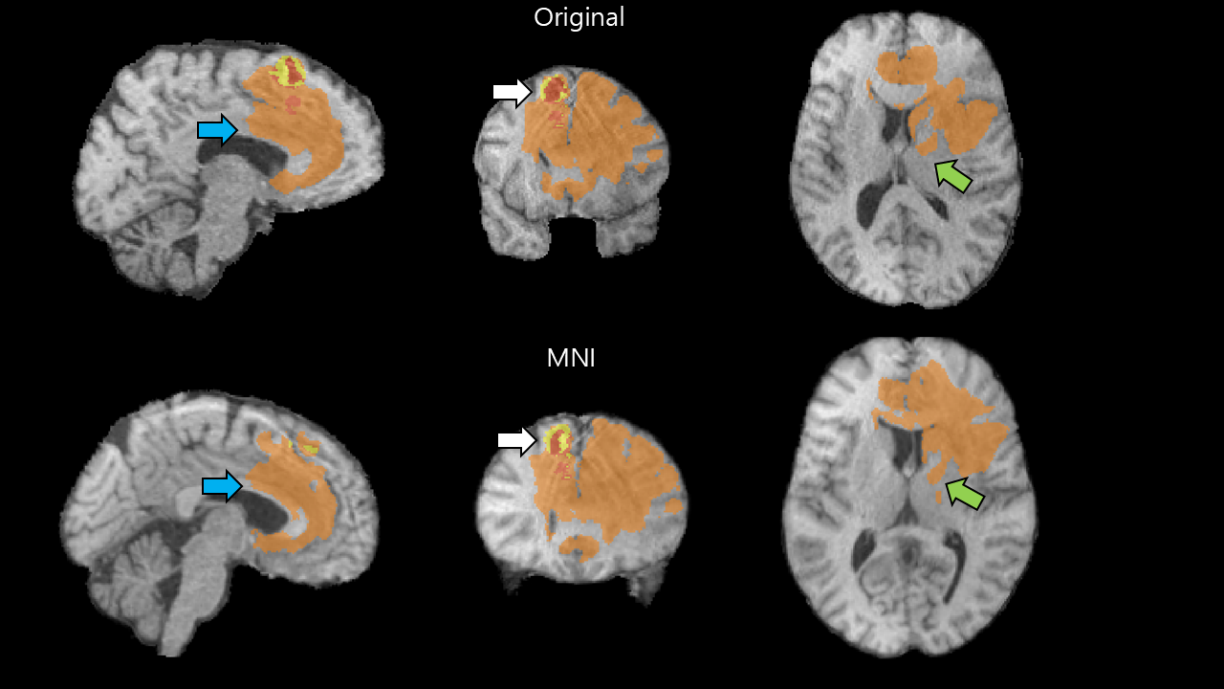


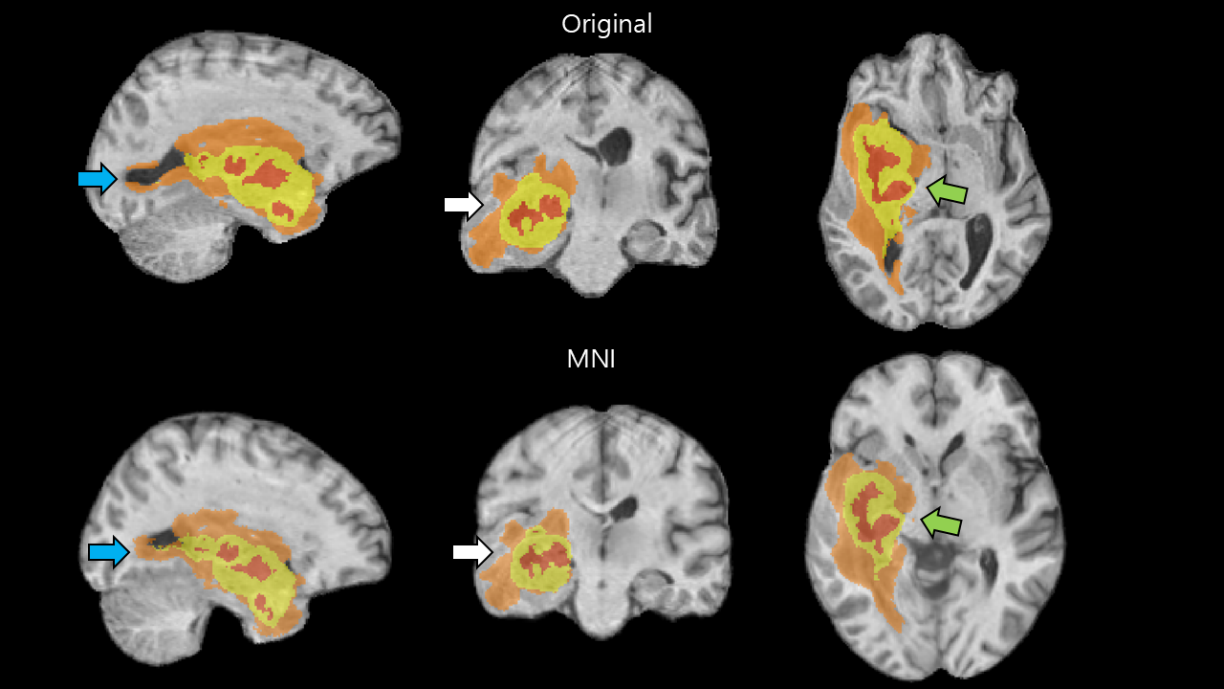


Figure S4. Result of sensitivity analysis using 5 mm radius sphere in aDTI-APLS calculation. Upper panels show the correlation between the two ROI definitions, while lower panels show the result of survival analysis. (A) UPENN and (B) UCSF cohorts.


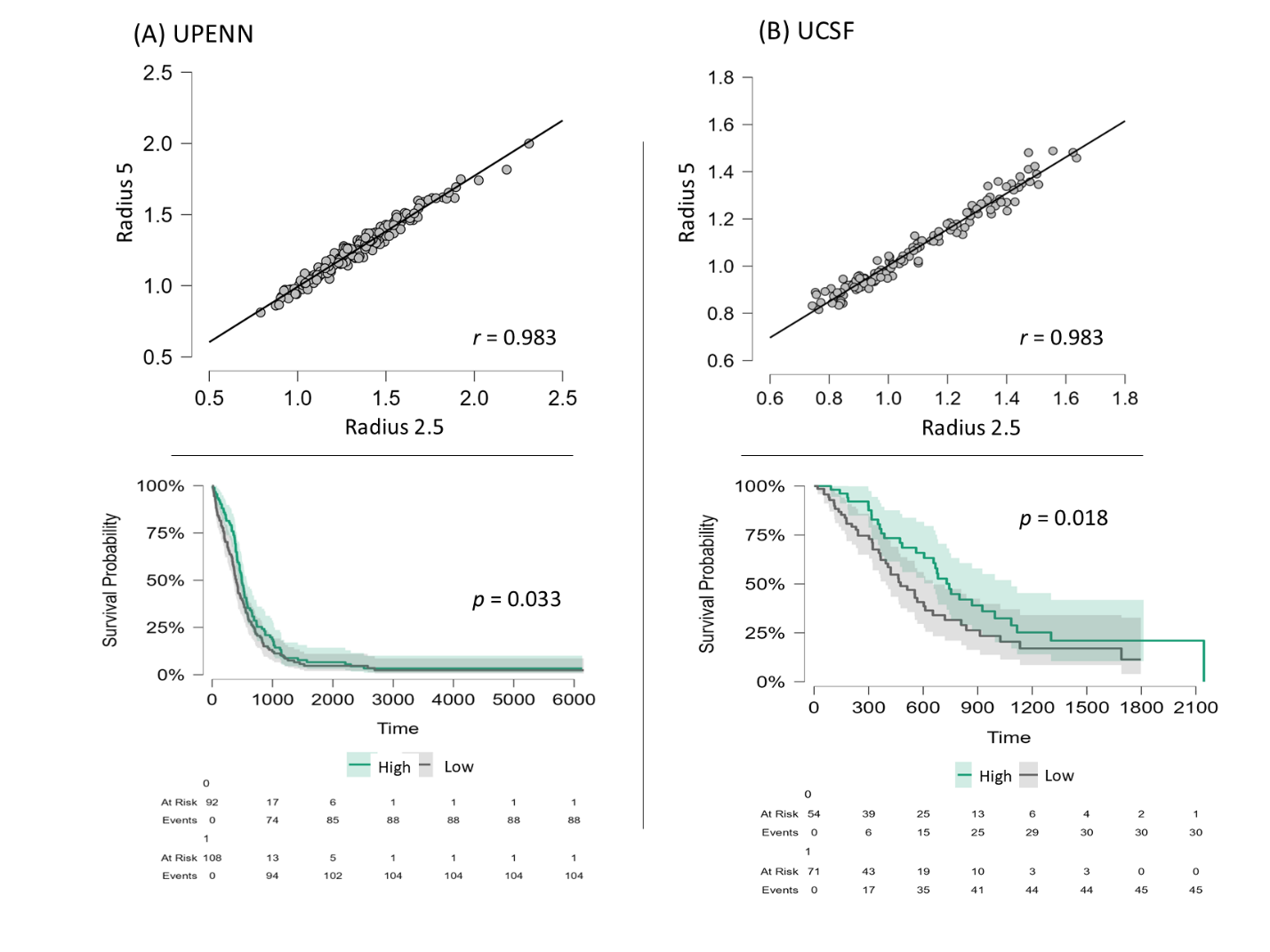

Supplement: Supplementary file 1 — Supplementary Material 1 [file 234_2026_3928_MOESM1_ESM.docx]
